# Variety of transversus thoracis muscle in relation to the internal thoracic artery: an autopsy study of 120 subjects

**DOI:** 10.1186/1749-8090-6-11

**Published:** 2011-01-27

**Authors:** Lazar Jelev, Stanislav Hristov, Wladimir Ovtscharoff

**Affiliations:** 1 Department of Anatomy, Histology and Embryology, Medical University of Sofia, blvd. Sv. Georgi Sofiiski 1, 1431 Sofia, Bulgaria; 2 Department of Forensic Medicine and Deontology, Medical University of Sofia, blvd. Sv. Georgi Sofiiski 1, 1431 Sofia, Bulgaria

## Abstract

**Background:**

The transversus thoracis muscle is a thin muscular layer on the inner surface of the anterior thoracic wall that is always in concern during harvesting of the internal thoracic artery. Because the muscle is poorly described in the surgical literature, the aim of the present study is to examine in details its variations.

**Methods:**

The data was obtained at standard autopsies of 120 Caucasian subjects (Bulgarians) of both sexes (97 males and 23 females), ranging in age from 18 to 91 years (mean age 52.8 ± 17.8 years). The transversus thoracis morphology was thoroughly examined on the inner surface of the chest plates collected after routine incisions.

**Results:**

An overall examination revealed that in majority of cases the transversus thoracis slips formed a complete muscular layer (left - 75.8%, right - 83.3%) or some of the slips (left - 22.5%, right - 15%) or all of them (left - 1.7%, right - 1.7%) were quite separated. Rarely (left - 3.3%, right - 5.8%), some fibrous slips of the transversus thoracis were noted. In 55.8% of the cases there was left/right muscle symmetry; 44.2% of the muscles were asymmetrical. Most commonly, the highest muscle attachment was to the second (left - 53.3%, right - 37.5%) or third rib (left - 29.2%, right - 46.7%). The sixth rib was the most common lowest attachment (left - 94.2%, right - 89.2%). Most frequently, the muscle was composed of four (left - 31.7%, right - 44.2%) or fifth slips (left - 53.3%, right - 40.8%).

**Conclusions:**

This study provides detailed basic information on the variety of the transversus thoracic muscle. It also defines the range of the clearly visible, uncovered by the muscle part of the internal thoracic artery and the completeness of the muscular layer over it. The knowledge of these peculiar muscle-arterial relations would definitely be beneficial to cardiac surgeon in performing fast and safe arterial harvesting.

## Background

The inner surface of the anterior thoracic wall is covered by a thin muscular layer - *transversus thoracis muscle *[1-4]. It has a close relation to the internal thoracic artery (ITA), which is now accepted as a superior graft for CABG surgery [1,2]. According to classical textbook descriptions [3,4], the fibers of transversus thoracis form four to five slips. Those arise from the xiphoid process, the inferior part of the body of sternum and the adjacent costal cartilages near their sternal ends, and directing supero-laterally they insert from the second to sixth costal cartilages. In literature it is also mentioned that the transversus thoracis shows variations in its attachments not only in different subjects, but also on the opposite sides of the same subject [4]. Consequently, that particular muscle was announced as the most variable in the human body [5]. Anatomists from the XIXth and the beginning of the XXth century have reported the variations of the transversus thoracis only qualitatively [6-8]. Some quantitative evaluation of the rib attachment level of the transverus thoracis slips have been presented only by Loth [9], Mory [10] and Satoh [11].

Surprisingly, in the surgical literature there is quite insufficient data about the most variable human muscle, which is currently having a role in CABG surgery. Acquiring detailed information about the transversus thoracis variations would provide basic anatomical information for the cardiac surgeons while performing ITA harvesting.

## Methods

The data presented here was gathered in the course of fresh cadaver autopsies carried out in the Department of Forensic Medicine at the Medical University of Sofia, Bulgaria. The medico-legal office and local Ethic Committee approved the study. During the last four years (2006-2009) a total of 120 Caucasian subjects (Bulgarians) of both sexes (97 males and 23 females), ranging in age from 18 to 91 years (mean age 52.8 ± 17.8 years) were examined. None of the autopsied persons had ever undergone any prior thoracic surgical procedure. A standard autopsy protocol was followed for each one of the bodies [12]. After initial midline incision on the anterior thoracic wall, the skin and the subcutaneous tissue were dissected back to expose the underlying muscles and bones. The sternoclavicular joints on both sides were identified and cut. With a bone saw the ribs were cut along the anterior axillary line and the chest plate containing the sternum, the medial part of the upper eight to nine ribs and the surrounding soft tissues was removed from the body. Afterwards, the fat tissue on the inner surface of the chest plate was carefully removed. That helped in observing and recording the characteristics of the transversus thoracis. The following data analysis was done over 240 thoracic halves - 120 left and 120 right ones.

## Results

**Figure 1:**
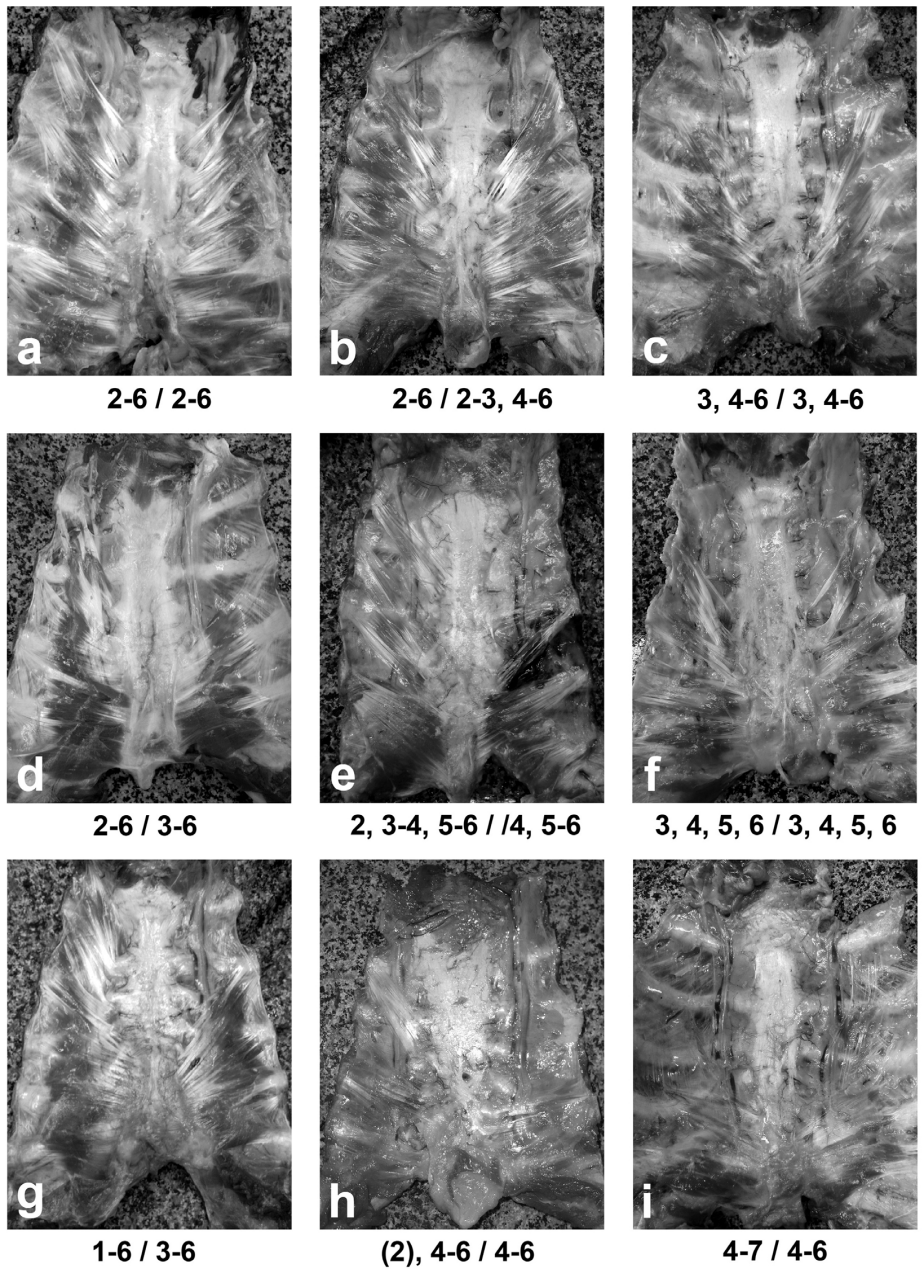
**Photographs of various forms of the transversus thoracis muscle explained by short digital formula (see in the text)**.

During this study a wide range of variations of the transversus thoracis muscle were recorded. Some examples are given in Figure 1a-i. To group and analyze these variations, however, seemed to be a difficult task. For clear and concise presence of the peculiar forms of the transversus thoracis and following data analysis, a short digital formula was used in each one of the cases. This formula represents the highest and lowest level of rib insertion of the transversus thoracis slips and also their separation on both sides. For example, one of the most commonly found muscle variants (Figure 1a) could be expressed as "2-6/2-6" which means "a complete layer of muscular slips attached from second to sixth ribs on both sides". A more complicated case such as "3, 4-6/3, 4-6" (Figure 1c) has the meaning of "a bilateral presence of a separate muscular slip to the third rib and complete layer of slips from fourth to sixth ribs". Rarely, a slip of the transversus thoracis may be completely fibrous but not muscular; in such cases the respective fibrous slip is given in parenthesis - "(2), 4-6/4-6" (Figure 1h).

**Figure 2:**
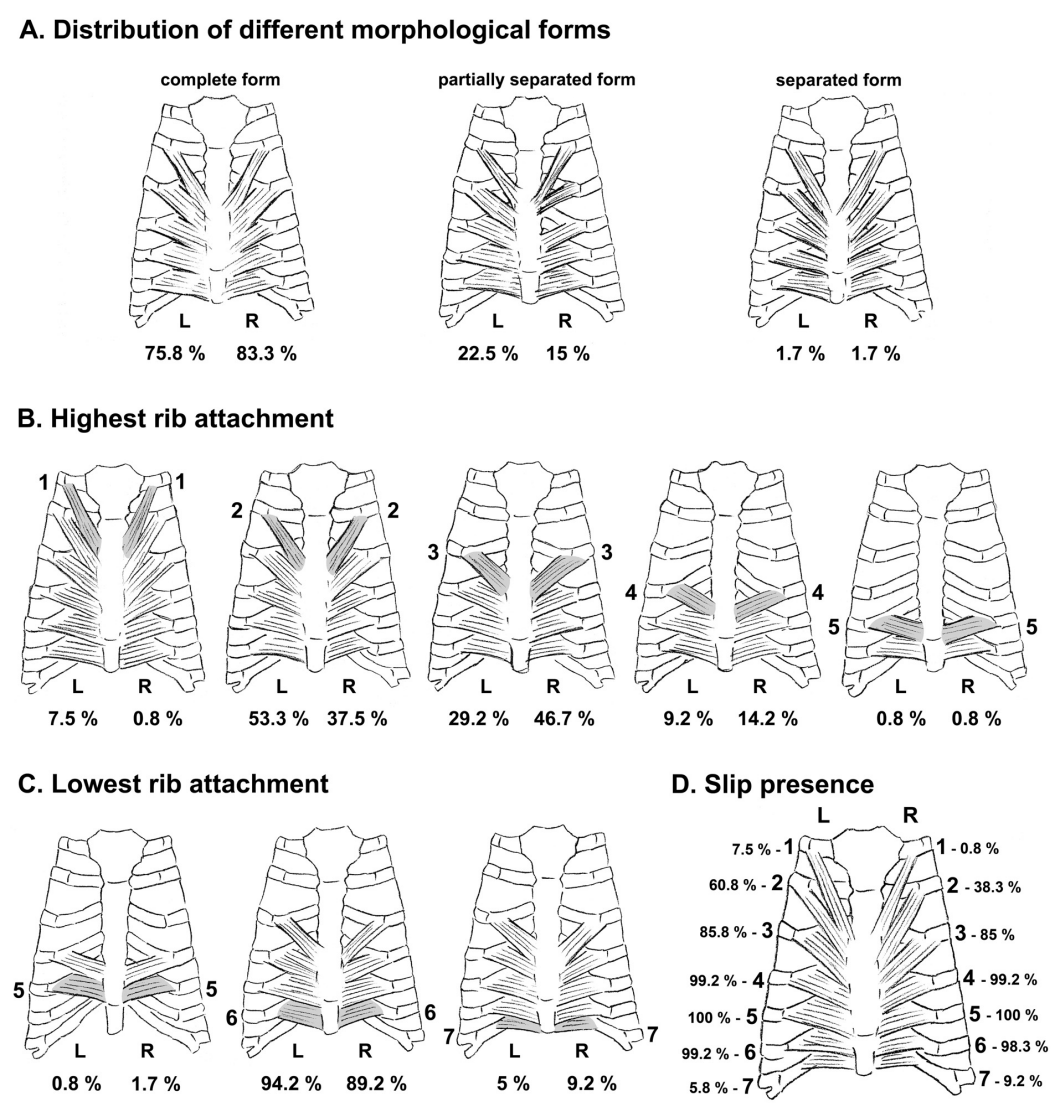
**Schemes showing numerical distribution of different characteristics of the transversus thoracis muscle**.

An overall examination of the morphology of the transversus thoracis has led us to defining three morphological forms (Figure 2.A). Usually, the fibers of the transversus thoracis formed a complete layer of slips between their rib attachments (Figure 1a,d,g), i.e. a complete muscular layer covered the ITA. In some cases, there were separation of upper muscular slips on either side (Figure 1b,c,e). As a rare instance, all of the transversus thoracis slips were separated from one another (Figure 1f). In the last two muscle forms, different in size and width muscular bridges along the ITA course could be observed. In small number of cases (3.3% on the left and 5.8% on the right side), some slips of the transversus thoracis, completely fibrous in nature, were noted (Figure 1h). Usually they were found in the upper part of the chest plate and were either connected to or separated from the muscular slips. Commonly, the slips of the transversus thoracis were arranged successively between their highest and lowest rib attachments. In 4.2% of the cases on the left side a missing slip to the third rib was observed (Figure 1h), consequently there was a cleft between the second and forth ribs. Analyzing the bilaterality of the transversus thoracis, it was established that in 55.8% of the cases there was symmetry between the left and right side muscles. However the remaining 44.2% of the muscles were asymmetrical.

Frequently, the highest rib attachment of the transversus thoracis slips (Figure 2B) was to the second or third rib with some differences between the left and right side. In minority of cases, the fourth rib was the highest level of slip attachment. The highest rib attachment was seldom observed at either first or fifth rib. Three successive ribs (fifth, sixth or seventh) served as a lowest rib attachment for the transversus thoracis slips (Figure 2C). The sixth rib was the most common level of attachment (94.2% on the left and 89.2% on the right side). Regarding the percentage of presence of every slip in the autopsy material, the slip to the fifth rib on both sides always existed. The slips to the fourth and sixth ribs were presented in most of cases (Figure 2D). The number of slips forming the transversus thoracis in our sample also varied. Between the highest and lowest rib attachments in vast majority of the cases, four or fifth slips were identified (table 1). The "scope" of the transversus thoracis on the inner thoracic wall (table 2) shows distribution of different muscle forms. Those are defined by combination of their highest and lowest rib attachments. On the left side, in higher percentage of the cases there was a muscle with formula 2-6. On the right side, muscles with formulas 2-6 and 3-6 were observed more often.

**Table 1:** Number of slips of the transversus thoracis muscle.

LEFT SIDE	RIGHT SIDE
**1**	SLIP

0.8%	-

**2**	SLIPS

-	2.5%

**3**	SLIPS

7.5%	10%

**4**	SLIPS

31.7%	44.2%

**5**	SLIPS

53.3%	40.8%

**6**	SLIPS

5.8%	2.5%

**7**	SLIPS

0.8%	-

**Table 2:** Scope of the transversus thoracis muscle on the inner thoracic wall.

		LEFT	SIDE			RIGHT	SIDE		
HIGHEST	**1-st**	-	6.7%	0.8%	-	0.8%	-	**1-st**	HIGHEST

RIB	**2-nd**	-	52.5%	0.8%	1.7%	35.8%	-	**2-nd**	RIB

	**3-rd**	-	27.5%	1.7%	5%	41.7%	-	**3-rd**	

	**4-th**	-	7.5%	1.7%	2.5%	10%	1.7%	**4-th**	

	**5-th**	0.8%	-	-	-	0.8%	-	**5-th**	

		**5-th**	**6-th**	**7-th**	**7-th**	**6-th**	**5-th**		

		LOWEST	RIB			LOWEST	RIB		

## Discussion

During the years, the term "transversus thoracis" has been used differently in the anatomical literature [13]. Recently [4], the muscle is also termed "triangularis sterni" and "sternocostalis". Those particular terms are important for the detailed literature examination of transversus thoracis variations. According to the classical works on the muscular variations by Macalister [6], Le Double [7] and Eisler [8], the following variations of the transversus thoracis have been described: complete absence; separation into distinct fascicles; presence of a slip attached from the second rib to forth rib cartilage and passing over the third rib; presence of a varying number of slips from six to two; presence of only one slip; continuation with the fibers of the transversus abdominis muscle; presence of a separate upper slip from the sternum to the second-rib cartilage. The majority of these variations we encountered in our autopsy series. The quantitative data on the transversus thoracis variations are scarce in literature. Data concerning the level of slip attachment and scope of the transversus thoracis, irrespective of the side examined, have been reported by Loth [9] in Poles and Mory [10] and Satoh [11] for Japanese population. A comparison between theirs and our results is shown in table 3. The second and third ribs seem to be the most common highest rib attachments of the transversus thoracis in Poles, Japanese and Bulgarians. The most frequent lowest rib attachment in three populations examined was the sixth rib, but in Bulgarians it occupies nearly 90% of the cases, compared to 76% in Poles and Japanese. Analyzing the population differences of the transversus thoracis scope reveals that forms 2-6 and 3-6 are present in the majority of cases. Besides transversus thoracis, the sternalis muscle and axillary arch (other common variant muscles of the thoracic wall) also possess a vast population variety [14-16].

**Table 3:** Comparison of the quantitative literature data concerning the transversus thoracis muscle.

*Author (year) *[Reference] Population examined	***Loth (1931) ***[9] Poles	***Mori (1964) ***[10] Japanese	***Satoh (1971) ***[11] Japanese	***Jelev et al. ***[present study] Bulgarians
Highest rib attachment				Left side Right side

1-st	9%	2.2%	12.5%	7.5% 0.8%

2-nd	42%	39.3%	58.3%	53.3% 37.5%

3-rd	40%	50%	18.8%	29.2% 46.7%

4-th	8%	7.4%	2.1%	9.2% 14.2%

5-th	1%	0.9%	-	0.8% 0.8%

Lowest rib attachment				

5-th	6%	16.1%	14.6%	0.8% 1.7%

6-th	76%	76.4%	68.8%	94.2% 89.2%

7-th	17%	7.4%	6.3%	5% 9.2%

Scope (most frequent forms)				

2-6	32%	27.7%	-	52.5% 35.8%

3-6	30%	39.3%	-	27.5% 41.7%

Regarding muscle function, transversus thoracis draws down the costal cartilages [3,4] and takes a stand on the expiration phase of breathing. Unlike dogs where the transversus thoracis is a primary muscle of breathing [17], in human this muscle is usually silent in supine posture [18]. In standing posture it contributes to the deflation of the rib cage during active expiration [19]. Our data reveals that in most of the cases the transversus thoracis is well developed bilaterally. However, the cases with weakly developed muscle are an evidence of secondary breathing role of the transversus thoracis in human.

The transversus thoracis variations represent theoretical interest with some practical significance. Usually during routine CABG surgery, the harvesting of ITA starts in the bare area on the inner thoracic wall, between the first rib and the highest transversus thoracis slips. At this location the artery is well detectable because it is covered only by the endothoracic fascia and the parietal pleura [2]. By describing the transverusus thoracis variations we provide characteristics of the possible size of this bare area. Usually it reaches second and third ribs and intercostal spaces; rarely it can be missing (the highest muscular attachment is to the 1-st rib) or spreads over almost entire course of the ITA (the highest muscular attachment is to the 5-th rib). On the left side, in 53.3% the bare area reaches below the second rib and intercostal space; in nearly half of the cases (46.7%) the right bare area is larger and reaches the level of third rib and intercostal space.

## Conclusions

The data from the present study adds some basic information concerning the surgical anatomy of the anterior thoracic wall. It describes the variety of the transversus thoracis muscle and its relation to the ITA. A complete muscular layer of significant size predominantly covers the artery. Rarely, this muscular layer is quite narrow or separate muscular slips bridge over the artery. Usually, the ITA is clearly visible under the endothoracic fascia and parietal pleura up to the second or third rib level. Sometimes, the artery may be uncovered or completely covered by the muscle fibers through its entire course. All this muscle-arterial relations, quite variable in nature, may be borne in mind during routine ITA harvesting and especially when using minimally invasive approaches and endoscopic harvesting techniques [20-26].

## Competing interests

The authors declare that they have no competing interests.

## Authors' contributions

LJ and WO carried out the study design, data analysis and writing, LJ and SH performed data collection, WO and SH made a critical review of the manuscript. All authors read and approved the final manuscript.
